# Diversity and Bioactive Potential of Actinobacteria Isolated from a Coastal Marine Sediment in Northern Portugal

**DOI:** 10.3390/microorganisms8111691

**Published:** 2020-10-30

**Authors:** Inês Ribeiro, Mariana Girão, Diogo A. M. Alexandrino, Tiago Ribeiro, Chiara Santos, Filipe Pereira, Ana P. Mucha, Ralph Urbatzka, Pedro N. Leão, Maria F. Carvalho

**Affiliations:** 1 Interdisciplinary Centre of Marine and Environmental Research, University of Porto, 4450-208 Porto, Portugal; iribeiro@ciimar.up.pt (I.R.); mariana.martins@ciimar.up.pt (M.G.); dalexandrino@ciimar.up.pt (D.A.M.A.); tiago.amribeiro8@gmail.com (T.R.); chiaragabriele5@gmail.com (C.S.); fpereirapt@gmail.com (F.P.); amucha@ciimar.up.pt (A.P.M.); rurbatzka@ciimar.up.pt (R.U.); pleao@ciimar.up.pt (P.N.L.); 2 Institute of Biomedical Sciences Abel Salazar, University of Porto, 4050-313 Porto, Portugal; 3 Faculty of Sciences, University of Porto, 4169-007 Porto, Portugal

**Keywords:** Actinobacteria, secondary metabolism, natural products, antimicrobial activity, cytotoxic activity, coastal sediment

## Abstract

Natural compounds have had increasing applications in the biotechnological sector, with a large fraction of these substances being channeled to the pharmaceutical industry due to their important pharmacological properties. The discovery of new bioactive molecules with novel mechanisms of action constitutes a promising solution for the design of alternative therapeutic solutions. Actinobacteria are a large group of morphologically and physiologically diverse bacteria well known for their production of biotechnologically relevant compounds. The Portuguese coast is scantly explored in terms of Actinobacteria diversity and respective bioactive potential, offering a good opportunity to find new Actinobacteria taxa and bioactive natural products. In this study, we investigated the Actinobacteria diversity associated with a sediment sample collected from the intertidal zone of a beach in northern Portugal, through a cultivation-dependent approach, and screened its antimicrobial and cytotoxic potential. A total of 52 Actinobacteria strains were recovered from the marine sediment, with the largest fraction of the isolates belonging to the genus *Micromonospora*. Bioactivity screening assays identified crude extracts of six *Streptomyces* strains active against *C. albicans*, exhibiting minimum inhibition concentration (MIC) values in the range of 3.90–125 μg mL^−1^. Twenty-five Actinobacteria crude extracts (obtained from strains of the genera *Micromonospora*, *Streptomyces* and *Actinomadura*) exhibited significant effects on the viability of at least one tested cancer cell line (breast ductal carcinoma T-47D and liver hepatocellular carcinoma HepG2). The Actinobacteria extracts demonstrating activity in the antimicrobial and/or cytotoxic assays were subjected to metabolomic analysis (Mass spectrometry (MS)-based dereplication and molecular networking analyses), indicating the presence of four clusters that may represent new natural products. The results obtained demonstrate the importance of bioprospecting underexplored environments, like the Portuguese coast, for enhancing the discovery of new natural products, and call attention to the relevance of preserving the natural genetic diversity of coastal environments.

## 1. Introduction

Multidrug-resistant bacterial infections and cancer are at the top of diseases for which novel therapies are required. The increase of antibiotic-resistant microorganisms is a serious public health problem that jeopardizes the effectiveness of current antimicrobial therapies, leading to a growing need of finding new therapeutic agents with different mechanisms of action [1,2]. Most anticancer drugs currently used to treat several types of cancer diseases cause undesirable side effects, damaging healthy cells and tissues while killing cancer cells, and for many types of cancer there is still no effective treatment available, it being urgent to discover new molecules with novel anticancer actions and less side effects [1].

Natural products (NP) represent a large and diverse family of chemicals produced by various living organisms and generally bear important biological properties [3]. These compounds have paramount importance for the pharmaceutical industry, illustrated by the fact that in the period between 1981 and 2019 more than 50% of all approved drugs were from natural origin or inspired by natural molecules [4]. In the last 30 years, more than 40,000 NPs with microbial origin have been characterized, the highest percentage of these being derived from bacteria [5].

The class Actinobacteria comprises a group of microorganisms highly prolific in the production of NP with several relevant bioactivities, being responsible for the production of about 45% of all known bioactive microbial metabolites [5]. Species affiliated with the genus *Streptomyces* are the main protagonists of the large repertoire of bioactive metabolites produced by Actinobacteria, contributing to the production of ca. 75% of these compounds [5,6]. Actinobacteria inhabiting terrestrial environments have been intensively prospected for the production of NP, leading to a reduction in the discovery of novel bioactive molecules from these microorganisms [7]. In the last years, a high interest in marine Actinobacteria has emerged, not only for their taxonomic diversity and ecological relevance, but also for their capacity to produce novel NP [8]. Oceans are home to a great diversity of microorganisms, including Actinobacteria, though a large majority of these are still unknown and unexplored [7]. The indigenous character of marine Actinobacteria has only recently been recognized and these microorganisms have proven to be an important source of bioactive compounds [9]. Examples include marinomycins, produced by a *Marinospora* sp., with anticancer and antibacterial activity, salinosporamide, derived from the species *Salinispora tropica* and a potent anticancer agent, and the antibiotic abyssomicin produced by *Verrucosispora maris* [7,9,10,11].

Marine sediments of the Atlantic ocean are very poorly explored in terms of NP-producing microorganisms [12] and this includes the vast marine environments of Portugal for which the associated Actinobacteria population is scarcely studied. Such underexplored environments offer an excellent opportunity to discover new microorganisms and NP [13].

In this study, a sediment sample collected from the intertidal zone of a beach located in Parque Natural do Litoral Norte, a 16 km protected area on the northern coast of Portugal classified as a Natural Park, was used to investigate the diversity of culturable Actinobacteria and the associated potential to produce compounds with antimicrobial and cytotoxic activities. This Natural Park was created in 2005 for the preservation of its natural, physical and scenic properties, and in particular for the preservation of the dune system. It consists of a strand of beaches and sand dunes that integrate reefs and other marine habitats. The marine zone of Parque Natural do Litoral Norte has a rocky substrate with outcrops and nutrient-rich cold waters and it is bathed by the Atlantic Ocean. The importance of the biological and habitat diversity of this region, and the fact that it is completely unexplored in terms of Actinobacteria diversity and respective biotechnological potential, makes Parque Natural do Litoral Norte an attractive spot for bioprospection studies.

## 2. Materials and Methods

### 2.1. Sample Collection

A sample of marine sediment was collected at a depth of 0.15 m in the intertidal zone of a sandy beach located in Parque Natural do Litoral Norte (Cepães beach), in northern Portugal (41°33′11.5″ N, 8°47′38.1″ W). The sample was transferred to a sterile plastic bag, transported to the laboratory in a cooling box and processed within an interval of two hours.

### 2.2. Isolation of Actinobacteria

For the isolation of Actinobacteria, the sediment sample was processed using three methods: dilution of 1 g of sediment in 9 mL of filtered seawater (method 1); dilution of 1 g of sediment in 9 mL of filtered seawater followed by incubation in a water bath at 60 °C for 10 min (method 2); dilution of 5 g of sediment in 15 mL of filtered seawater and incubation with 20 mgL^−1^ of nalidixic acid (AppliChem, Darmstadt, Germany) and cycloheximide (Sigma-Aldrich, MO, United States) at 28 °C for 30 min (method 3). The resultant sediment samples were serially ten-fold diluted until 10^−5^. An aliquot of 100 µL of each dilution was spread over the surface of the following selective isolation media: (1) M3 agar (per liter of distilled water): 0.466 g of KH_2_PO_4_, 0.732 g of Na_2_HPO_4_, 0.10 g of KNO_3_, 0.29 g of NaCl, 0.10 g of MgSO_4_.7H_2_O, 0.02 g of CaCO_3_, 200 μg of FeSO_4_.7H_2_0, 180 μg of ZnSO_4_.7H_2_O, 15 μg of MnSO_4_.4H_2_0, 4 mg of thiamine HCl (Vitamin B1) and 17 g of agar; (2) nutrient-poor sediment extract (NPS) (per liter of seawater): 100 mL of marine sediment extract (obtained by washing 900 mL of sediments with 500 mL of seawater) and 17 g of agar; (3) starch-casein-nitrate agar (SNC) (per liter of distilled water): 10 g of soluble starch, 0.3 g of casein, 2 g of K2HPO4, 2 g of KNO_3_, 2 g of NaCl, 0.05 g of MgSO_4_.7H_2_O, 0.02 g of CaCO_3_, 0.01 g of FeSO_4_.7H_2_0 and 17 g of agar. All media were supplemented with cycloheximide (50 mgL^−1^; Sigma-Aldrich, MO, United States) and streptomycin (50 mg L^−1^; Sigma-Aldrich, MO, United States) in order to inhibit the growth of fungi and Gram-negative bacteria. The plates were incubated up to six weeks at a temperature of 28 °C (INCU-Line, VWR, PA, United States). Throughout the incubation period, plates were periodically observed and colonies with different morphological characteristics were picked and restreaked in the same agar medium until pure colonies were obtained. Each isolate was cryopreserved at −80 °C in 30% (*v*/*v*) glycerol. Biomass for cryopreservation was obtained by growing each isolate in liquid ISP2 (composition per liter of seawater: 4 g of yeast extract, 10 g of malt extract and 4 g of glucose) or marine broth (Laboratorios Conda, Madrid, Spain), supplemented with the same antibiotics as the other media.

### 2.3. Identification of the Isolated Strains by 16S rRNA Gene Sequencing

Biomass for DNA extraction was obtained by growing each isolate in 5 mL of ISP2 medium or marine broth (Table S1) at 25 °C, 100 rpm, for one week, followed by centrifugation (MicroStar 12, VWR, Radnor, PA, United States) of 2 mL broth at 7000× *g* for 5 min. Genomic DNA was extracted from all isolates though the phenol-chloroform method, as described elsewhere [14].

The 16S rRNA gene was amplified by polymerase chain reaction (PCR), using the universal primers 27F (5′-GAGTTTGATCCTGGCTCAG-3′) and 1492R (5′-TACGGYTACCTTGTTACGACTT-3′) [15]. The PCR mixture (total volume of 10 μL) contained 5 μL of Taq PCR Master Mix (Qiagen, Valencia, CA, USA), 1 μL of primer 27F (2 µM), 1 μL of primer 1492R (2 µM) and 3 μL of DNA template. PCR conditions were as follows: initial denaturation at 95 °C for 15 min, followed by 30 cycles at 94 °C for 30 s, 48 °C for 90 s, 72 °C for 90 s and a final extension at 72 °C for 10 min. PCR products were separated on a 1.5% agarose gel containing SYBR Safe (ThermoFisher Scientific, NY, USA), at 150 V for 30 min. Purification and sequencing of the amplified DNA fragment was performed by GATC Biotech (European Genome and Diagnostics Centre, Constance, Germany). The obtained 16S rRNA gene sequences were analyzed using the Geneious software, version 11.1.4. (Biomatters, Auckland, New Zealand) and the resulting consensus sequences were compared to the nucleotide collection (*nr*/*nt*) database from NCBI BLAST using the blastn algorithm (https://blast.ncbi.nlm.nih.gov/Blast.cgi) and confirmed by additional comparison with the databases EzTaxon (http://www.ezbiocloud.net/) and Ribosomal Database Project (https://rdp.cme.msu.edu/index.jsp). The 16S rRNA gene sequences of the isolated Actinobacteria strains were deposited in GenBank (NCBI, Bethesda, MD, USA) (Table S1).

A phylogenetic tree was performed by aligning the sequences of the obtained Actinobacteria isolates and the five closest neighbor sequences in Genbank for each isolate, using the MUSCLE alignment tool from the Geneious software. The alignment was used to generate a maximum likelihood phylogenetic tree with 1000 bootstraps based on the Tamura-Nei model. The phylogenetic tree was performed using the Molecular Evolutionary Genetics Analysis program Version 7.0 [16].

### 2.4. Preparation of Crude Extracts

Crude extracts were prepared by growing each isolate in 250 mL Erlenmeyer flasks containing 100 mL of ISP2 medium or marine broth (without the addition of cycloheximide and streptomycin) (Table S1). The flasks were incubated in a rotatory incubator (Model 210, Comecta SA, Barcelona, Spain) at 28 °C, 100 rpm, in the dark. Cultures were grown for 1–2 weeks, depending on their growth rate, after which 1.5 g of Amberlite^®^ XAD16N resin (Sigma-Aldrich, MO, USA) was added to the cultures and left to incubate for an additional week. Cultures were extracted twice with methanol/acetone 1:1 (*v*/*v*). The organic layer was dried in a rotary evaporator and the resulting extract was dissolved in dimethyl sulfoxide (DMSO, ≥99.9%, Sigma-Aldrich, MO, USA) to obtain stock solutions with final concentrations of 3.0 mg mL^−1^ and 1.0 mg mL^−1^, to be tested in the bioactivity assays.

### 2.5. Antimicrobial Bioactivity Screening

The screening of the Actinobacteria extracts for antimicrobial activity was performed using the agar-based disk diffusion method. The extracts were tested against five reference microorganisms: *Staphylococcus aureus* (ATCC 29213), *Bacillus subtilis* (ATCC 6633), *Escherichia coli* (ATCC 25922), *Salmonella typhimurium* (ATCC 25241) and *Candida albicans* (ATCC 10231). The bacterial strains were grown in Mueller-Hinton agar (MH) (Liofilchem, Roseto d. Abruzzi, Italy) and the yeast, *C. albicans*, was grown in Sabouraud dextrose agar (SD) (Liofilchem, Roseto d. Abruzzi, Italy). Colonies of the reference organisms were suspended in the corresponding medium broth and the turbidity of the cultures was adjusted to 0.5 McFarland standard (OD625 = 0.08−0.13). These standardized suspensions were used to seed MH (for the bacterial strains) or SD (for *C. albicans*) agar plates, by evenly streaking the plates with a swab dipped in the inoculum cultures. Blank paper discs (6 mm in diameter; Oxoid Limited, Hampshire, UK) were placed on the surface of the inoculated plates and loaded with 15 μL of each extract at a concentration of 1.0 mg mL^−1^. Negative controls consisted of loading the paper disks with 15 μL of DMSO, and positive controls consisted of loading the disks with 15 μL of enrofloxacin (1.0 mg mL^−1^; Sigma-Aldrich, MO, USA) for the bacterial strains, and 15 μL of nystatin (1.0 mg mL^−1^; Sigma-Aldrich, MO, USA) for *C. albicans*. Agar plates were incubated at 37 °C for 24 h, after which the presence of an inhibition zone was inspected, and the respective diameter was measured. Each extract was tested once in two independent experiments.

Minimum inhibitory concentration (MIC) was also determined for extracts exhibiting antimicrobial activity in the agar-based disk diffusion method. Inoculum suspensions of reference microorganisms were prepared as described above. Stock solutions of extracts were prepared in the appropriate medium broth (according to the reference microorganism) at a concentration of 500 µg mL^−1^ and ten two-fold dilutions using the same medium were successively performed from these stocks, resulting in extracts concentrations ranging from 250 to 0.487 µg mL^−1^. The assay was performed in 96 well plates to which 50 μL of microbial inoculum (diluted 1:100) and 50 μL of each extract dilution were added to each well. A positive growth control consisted of 50 μL of microbial inoculum and 50 μL of medium broth, and a negative growth control consisted of 100 μL of medium broth. MIC was determined by spectrophotometric analysis (625 nm; model V-1200, VWR, Pennsylvania, PA, USA), after 18 h of incubation at 37 °C. For each extract, MIC was determined in triplicate in two independent experiments.

### 2.6. Cytotoxic Bioactivity Screening

The Actinobacteria extracts were tested against two cancer cell lines: breast ductal carcinoma (T-47D) and liver hepatocellular carcinoma (HepG2), both from Sigma-Aldrich (St. Louis, MO, USA). Cell lines were grown in Dubelco’s modified eagle medium (DMEM; Gibco, Thermo Fischer Scientific, Waltham, MA, USA) supplemented with 10% (*v*/*v*) fetal bovine serum (Biochrom, Berlin, Germany), 1% (*v*/*v*) penicillin/streptomycin (Biochrom, Berlin, Germany) at 100 IU mL^−1^ and 10 mg mL^−1^, respectively, and 0.1% (*v*/*v*) amphotericin (GE Healthcare, Little Chafont, UK). The cells were incubated at 37 °C in a humidified atmosphere containing 5% of CO2 (Biosystem *w*/*o* Controller, Cimarec™, Thermo Fischer Scientific, MA, USA).

Cell viability was assessed by the MTT (3-(4,5-dimethylthiazol-2-yl)-2,5-diphenyltetrazolium bromide) assay. The cells were seeded in 96-well plates at a density of 6.6×104 cells mL^−1^. After 24 h, cells were exposed to the extracts at a final concentration of 15 µg mL^−1^. Solvent and positive controls consisted in of 0.5% DMSO and 20% DMSO, respectively. Cell viability was assessed at 24 and 48 h by adding MTT at a final concentration of 0.2 mg mL^−1^ (Sigma-Aldrich, MO, USA) and incubating for 4 h at 37 °C. The medium was then removed and 100 μL of DMSO were added per well, after which the absorbance was read at 570 nm (Synergy HTX, Biotek, Winooski, VT, USA). Cellular viability was expressed as a percentage relative to the solvent control. Each extract was tested in triplicate in two independent assays. The extracts exhibiting cytotoxic activity in at least one cancer cell line were also tested on a nontumour cell line (human brain capillary endothelial cells, hCMEC/D3, kindly donated by Dr. P. O. Courad, INSERM, France), to assess general cytotoxicity, following the same procedure described above.

### 2.7. Statistical Analysis

Results from the cytotoxic activity assay, a total of nine replicates, were statistically tested for significant differences compared to the solvent control. The significance level was established for all tests at *p* < 0.05. The Kolmogorov Smirnov test was used to verify normality distribution of data, and Barthlett’s test for equal variances. For parametric data, one-way ANOVA followed by Dunnet’s post hoc test was applied. If the data did not meet criteria for one-way ANOVA, data were square root transformed and retested for normality and equal variances. For nonparametric data, the Kruskal-Wallis test was used followed by Dunn’s multiple comparison test.

### 2.8. Dereplication and Molecular Network Analysis

Twenty-six extracts, which demonstrated activity in the antimicrobial assays and/or were able to inhibit cell viability of the tested cell lines, were selected for metabolomic analysis, namely MS-based dereplication and molecular networking analyses. Crude extracts resuspended in methanol (2 mg mL^−1^) were analyzed by liquid chromatography-high resolution electrospray ionization tandem mass spectrometry (LC-HRESIMS/MS). This analysis was performed on a Dionex Ultimate 3000 HPLC connected to a qExactive focus mass spectrometer controlled by XCalibur 4.1 software (Thermo Fisher Scientific, Waltham, MA, USA). The chromatographic step was carried out in an ACE UltraCore 2.5 Super C18 column (75 mm × 2.1 mm, Advanced Chromatography Technologies, Aberdeen, United Kingdom). Ten microliters of each extract were injected and separated using a gradient from 99.5% eluent A (95% water, 5% methanol, 0.1% formic acid, *v*/*v*) and 0.5% eluent B (95% isopropanol, 5% methanol, 0.1% formic acid, *v*/*v*) to 10% eluent A and 90 % eluent B, over 9.5 min and then maintained for six min before returning to initial conditions. The UV absorbance of the eluate was measured at 254 nm and full MS scans, at a resolution of 70,000 full width at half maximum (FWHM) (range of 150–2000 *m*/*z*), followed by data dependent MS2 (ddMS2, Discovery mode) scans at the resolution of 17,500 FWHM (isolation window used was 3.0 amu and normalized collision energy was 35) were carried out. The raw data obtained from the dereplication of the extracts was converted to the mzML format and submitted to the dereplication workflow of the Global Natural Products Social Molecular Networking platform (GNPS) [17]. Extracts that did not show relevant hits in the dereplication analysis that could explain the activities obtained were then submitted to the GNPS data analysis workflow applying default parameters (except precursor ion mass tolerance, which was set to 0.01 Da and fragment ion mass tolerance, which was set at 0.04 Da, to consider high resolution data). The obtained molecular network was loaded onto Cytoscape v3.6.1 [18] to visualize the chemical similarities between metabolites and the distribution of *m*/*z* clusters among different samples.

We focused on *m*/*z* clusters that were unique to a particular strain and manually checked the corresponding liquid chromatography-high resolution electrospray ionization mass spectrometry LC-HRESIMS data (isotope clusters, adducts) to determine the mass of each target metabolite. These presumed compound masses were submitted to three additional dereplication analyses: Insilico Peptidic Natural Product Dereplicator [19] with default parameters, excluding ion mass tolerance precursor (0.01 Da) and fragment ion mass tolerance (0.04 Da); and by searching the probable accurate mass in the Dictionary of NP (version 27.1, CRC Press, Abingdon, UK) and the NP atlas database [20]. When hits from compounds produced by Actinobacteria or other bacteria were obtained, literature searches were conducted to attempt to determine with higher confidence (through MS/MS and bioactivity data) the putative identity of the target compounds.

## 3. Results

### 3.1. Actinobacteria Isolated from Marine Sediment

**Figure 1:**
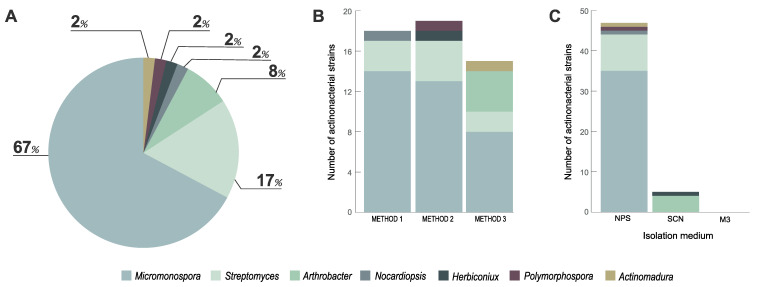
Distribution, in terms of genera, of the Actinobacteria strains recovered from the marine sediment collected at Parque Natural do Litoral Norte, northern Portugal, according to abundance, treatment methods and selective culture media used. (**A**) Percentage of recovered isolates belonging to indicated genera; (**B**) number of isolates affiliated with indicated genera recovered from the three treatment methods employed (Method 1, No treatment; Method 2, Water bath at 60 °C for 5 min; Method 3, Antibiotics (20 ppm) and water bath at 28 °C for 30 min); (**C**) number of isolates belonging to indicated genera recovered from to the three selective culture media used.

The isolation scheme employed in this study led to the recovery of 52 morphologically distinct Actinobacteria isolates from the studied coastal sediment, after an incubation period of six weeks. Many of these isolates exhibited traits characteristic of Actinobacteria, such as colonies with a leather texture and growing inside the agar, slow growth, production of hyphae or spores and production of pigments that in some cases changed the color of the medium. The taxonomic identification of these isolates is presented in Table S1. Analysis of the obtained taxonomies revealed that all treatments and culture media used in this study, except for medium M3, led to the isolation of Actinobacteria (Figure 1).

**Figure 2:**
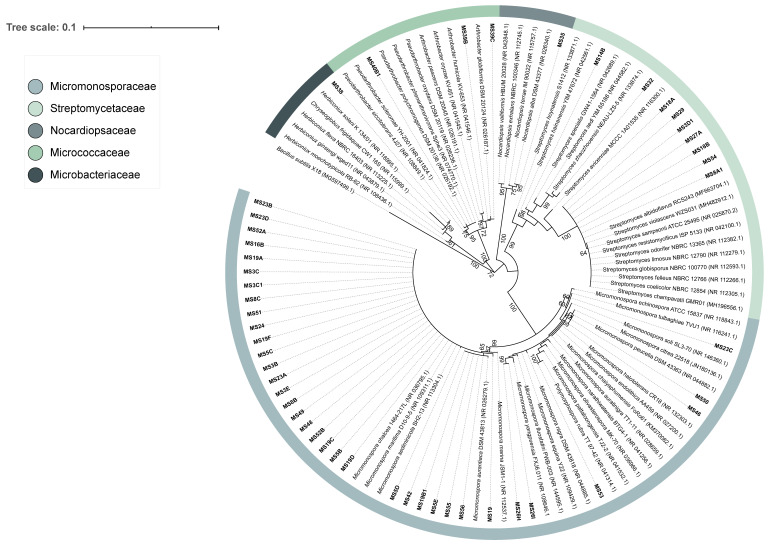
Phylogenetic tree of the Actinobacteria strains isolated from coastal marine sediment and their GenBank nearest neighbours. Maximum likelihood phylogenetic tree was performed in MEGA 7 using 107 sequences with 1090 bp. The phylogeny test used was the bootstrap method with 1000 replications. Bootstrap values shown at nodes support the branching order of the tree (values below 60% are not shown). The GenBank accession numbers are indicated in parenthesis. *Bacillus subtilis* X-18 was used as an outgroup. Scale bar corresponds to 0.1 substitutions per nucleotide position. The sequences of strains MS40A and MS58, assigned to the family Thermomonosporaceae, were not included in the tree as their length was <900 bp.

These isolates were distributed by six families (Microbacteriaceae, Micrococcaceae, Micromonosporaceae, Nocardiopsaceae, Streptomycetaceae and Thermomonosporaceae) and seven genera (*Arthrobacter*, *Actinomadura*, *Herbiconiux*, *Micromonospora*, *Nocardiopsis*, *Polymorphospora* and *Streptomyces*), with the largest fraction of the isolates being assigned to the genera *Micromonospora* and *Streptomyces* (Figure 1A and Figure 2). From the analysis of Figure 1B, it is apparent that treatment methods 1 and 2 allowed a similar recovery of strains of these genera. Method 3 led to the isolation of a lower number of *Micromonospora* and *Streptomyces* strains but several *Arthrobacter* isolates were obtained with this treatment (Figure 1B). The vast majority of the Actinobacteria isolates obtained were recovered from the culture medium NPS, with SCN retrieving a small fraction of these microorganisms and M3 medium showing no growth of Actinobacteria (Figure 1C).

Evolutionary relationships of the isolated Actinobacteria strains can be observed in the 16S rRNA gene phylogenetic tree presented in Figure 2. The different treatment methods and culture media employed for the isolation of Actinobacteria seem to have selected various isolates of the same species as many of them were found to group very closely, especially those affiliated with the genus *Micromonospora*. Phylogenetic analysis of the isolates assigned to the family Streptomycetaceae showed that the Actinobacteria strain MS14B formed a separate branch in the phylogenetic tree (Figure 2). This isolate is affiliated with the genus *Streptomyces* and has a similarity percentage of 98.13% with its closest neighbor in GenBank database (*Streptomyces hainanensis* strain YIM 47672), possibly constituting a new taxon given the similarity threshold for a new species of 98.7% [21].

### 3.2. Bioactivity Screening

The bioactive potential of all isolated Actinobacteria strains was investigated by screening crude extracts obtained from liquid cultures of these isolates for antimicrobial and cytotoxic activities. The results presented in Table 1 show that crude extracts of six *Streptomyces* isolates (strains MS8A1, MS18A, MS18B, MS27A, MS29 and MS54) exhibited antimicrobial activity against *C. albicans*, producing diameter halos between 8 and 25 mm when tested at 1.0 mg mL^−1^. Determination of MIC values for these extracts revealed biological activity against *C. albicans* in the range of 3.90–125 μg mL^−1^, with strain MS18B presenting the lowest MIC value (3.90 μL mL^−1^).

**Figure 3:**
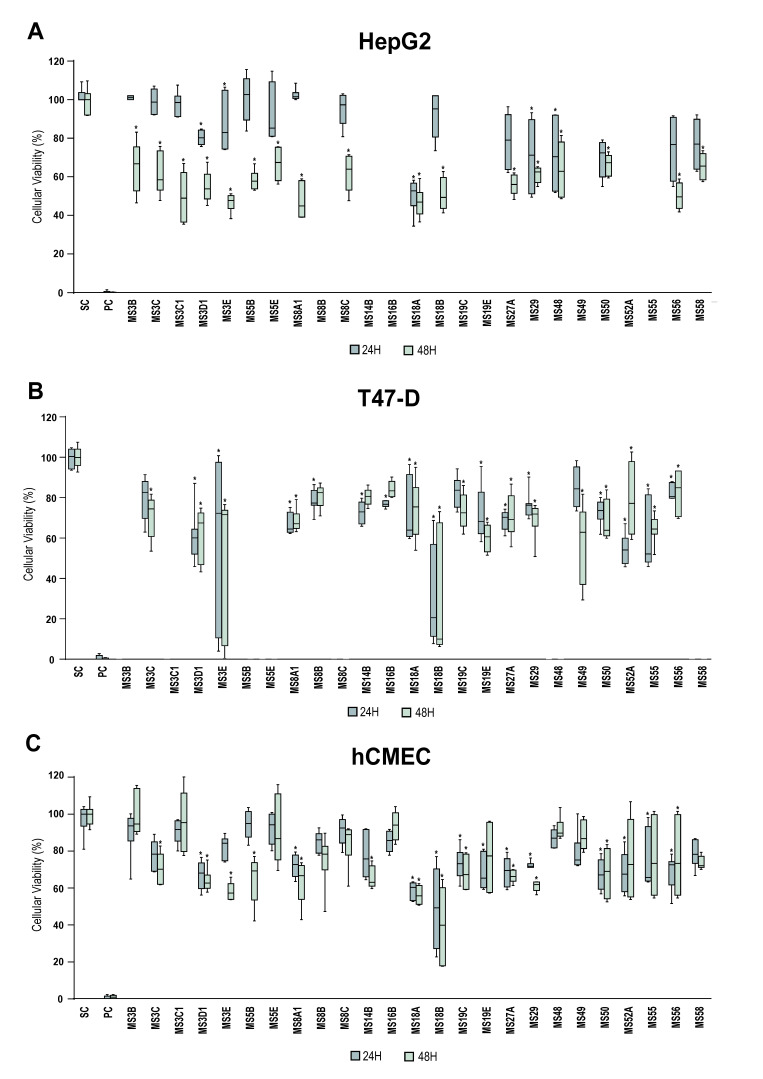
Cytotoxic activity of crude extracts of Actinobacteria strains isolated in this study, tested at 3.0 mg mL^−1^. The effects on the cellular viability of (**A**) liver cancer HepG2 and (**B**) breast carcinoma T-47D cancer cell lines, and (**C**) a nontumor hCMEC/D3 cell line (human brain capillary endothelial cells) are shown after 24 and 48 h of exposure The graphs relative to T47D and HepG2 cell lines only show extracts with significant differences. PC and SC denote positive and solvent controls, respectively. Values are presented as mean ± standard deviation from two independent assays conducted in triplicate and significant differences compared to the solvent control are annotated with asterisks in the graphs (* *p* < 0.05).

Crude extracts of 25 Actinobacteria isolates showed statistically significant cytotoxic activity against at least one of the cancer cell lines tested (Figure 3). Extracts from strains MS3C, MS3E, MS5B, MS48, MS50, MS56 (affiliated with the genus *Micromonospora*), MS58 (assigned to the genus *Actinomadura*) and MS3C1, MS3D1 MS8A1, MS18A, MS18B, MS27A, MS29 (all Streptomyces) caused a decrease in the viability of HepG2 cells of more than 40% (Figure 3A). Some of these crude extracts, namely from strains MS3E and MS18B, had the same effect in the T47-D (Figure 3B) and hCMEC/D3 cell lines (Figure 3C). The results showed that the majority of the tested crude extracts had a general cytotoxic effect, as they affected the viability of both cancer and normal cell lines. Interestingly, extracts from strains MS3B, MS3C1, MS5E, MS8C, MS48 and MS58 showed activity only on cancer cells, uniquely affecting the viability of the HepG2 cell line (Figure 3A). MS3C1 is clearly the most interesting extract, reducing 50% of HepG2 viability (Figure 3A), but not the normal cell line hCMEC/D3 (Figure 3C). Similarly, extracts from strains MS8B, MS16B and MS49 (all *Micromonospora*) had an identical effect on the T47-D cell line, selectively reducing its viability by more than 20% (Figure 3B).

**Figure 4:**
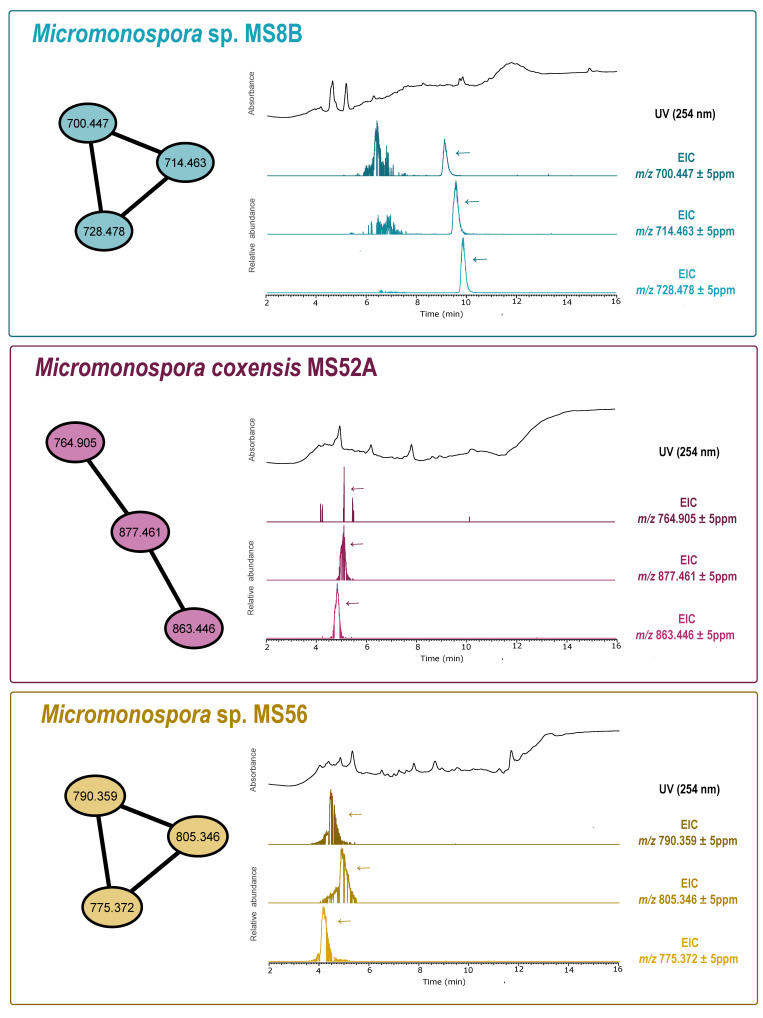
Analysis of the GNPS-generated molecular network LC-HRESI MS/MS of Actinobacteria crude extracts that showed bioactivity. The extracts obtained from one *Micromonospora coxensis* (MS52A) and two *Micromonospora* sp. (MS8B and MS56) exhibited exclusive clusters for each strain (represented by connected ellipses and marked with the corresponding *m*/*z* value for the parental ion) that did not match any entries in the databases used for dereplication. For each cluster we show the respective UV chromatogram and extracted ion chromatograms (EICs). Arrows depict peaks corresponding to the highlighted GNPS features.

To clarify whether the observed activity profiles were associated with known secondary metabolites, or, conversely, could be indicative of new natural products, a dereplication analysis was performed on 26 extracts that showed antimicrobial and/or cytotoxic activities. Seven of the tested extracts contained in their composition one or more members of the antimycin and nocardamine class (Table S2). The remaining 19 that did not show hits in the GNPS data analysis workflow that could explain the bioactivities obtained, were selected to build a molecular network using GNPS. The resulting network had 11 clusters belonging only to a single extract, which we defined as a key criterion chosen to increase the chance of a cluster corresponding to a previously unreported compound and, therefore, to undergo further dereplication using more comprehensive, non-MS/MS based databases. Six of such clusters were found in the extracts obtained from *Micromonospora* sp., five of which belonged to strain MS8B and another to strain MS56. Four out of the 11 clusters came from extracts of *Micromonospora coxensis*, with two belonging to strain MS5B and one to strain MS52A. Finally, two more single-strain clusters were found in the extracts of the strains *Streptomyces* sp. MS14B and *Micromonospora aurantiaca* MS55. Each of these 11 clusters underwent three additional dereplication steps (Insilico Peptidic Natural Product Dereplicator and by searching the probable accurate mass in the Dictionary of NP (version 27.1) and NP atlas database). For seven clusters, several hits from Actinobacteria, or other bacteria matching the accurate masses, were obtained that could explain the bioactivities presented in the different extracts (Figure S1). For the remaining four clusters, (one from *Micromonospora coxensis* MS52, two from *Micromonospora* sp. MS8B and one from *Micromonospora* sp. MS56) hits were found in the dereplication, but after comparing the MS2 spectra of the cluster mass and the corresponding hit compound, they were found not to be similar. Hence, such clusters can correspond to novel metabolites from this genus. The chromatographic peaks corresponding to the most abundant m/z values in each cluster revealed that they are likely to be produced in sufficient amounts, under the employed culture conditions, to enable isolation of the respective compounds after scaling-up (Figure 4). However, only for one cluster from *Micromonospora* sp. MS8B, the chromatographic peaks did not show sufficient abundance and, therefore, its cluster is not represented in Figure 4.

## 4. Discussion

In this study, we analyzed Actinobacteria associated with a marine sediment sample, collected from Cepães beach in Parque Natural do Litoral Norte, through culture-dependent methods. Fifty-two Actinobacteria isolates distributed by seven genera were retrieved from the coastal marine sediment, with the largest fraction of these isolates belonging to the genera *Micromonospora* and *Streptomyces*. The predominance of these genera in marine sediments has been described in other studies. Prieto-Davó et al. [12] identified 24 Actinobacteria genera in 198 marine sediments collected in various locations of Madeira archipelago and concluded that *Streptomyces*, *Actinomadura* and *Micromonospora* were the most abundant genera. Sediments collected in the North Sea and Chesapeake Bay, USA, were found to be rich in *Streptomyces*, *Nocardia*, *Micromonospora* and *Microbispora* species [22,23]. The genera *Micromonospora* and *Streptomyces* were found to be the most dominant in sediments collected from Trondheim Fjord, Norway [24] and marine sediments sampled from intertidal and subtidal zones around Bonne Bay, New Brunswick, Canada, were reported to be rich in the genera *Micromonospora*, *Nocardia*, *Nocardiopsis*, *Pseudonocardia* and *Streptomyces* [1,25].

The methods used in this study for treating the marine sediment sample have been successfully used for the isolation of Actinobacteria. Xiong et al. [26] employed seven different pretreatments, including incubation at 60 °C for 10 min (method 2 in this study) and incubation with nalidixic acid and cycloheximide at 28 °C for 30 min (method 3 in this study), for the isolation of Actinobacteria from various marine sediments collected from the Yellow Sea. The authors were able to isolate a high number of Actinobacteria strains (>600) mostly affiliated with the genera *Micromonospora*, *Kocuria*, *Nocardiopsis*, *Saccharomonospora* and *Streptomyces*. Abdelfattah et al. [27] recovered twenty-seven *Streptomyces* and *Nocardiopsis* species from marine sediments collected from the Red Sea coast by incubating the samples in a water bath at 60 °C for 10 min (method 2 in this study) and using the selective media humic-acid vitamin agar (HVA) and SCN.

The isolation of Actinobacteria from various marine sources has been shown to be more efficient when using culture media with low nutrient concentrations that better simulate the nutritional condition of marine environments and avoid the development of fast-growing microorganisms [28,29,30]. As such, we selected NPS medium as a nutrient-poor isolation medium that was formulated only with nutrients obtained from the same habitat of the isolated microorganisms and, in fact, this was the medium with the highest efficiency, leading to the largest recovery of Actinobacteria isolates (47 out of 52). Jensen et al. [30] recovered a high number of Actinobacteria isolates, belonging to *Micromonospora*, *Streptomyces* and *Actinomadura* genera, from a marine sediment collected in the island of Guam, Micronesia, USA, using this same medium. Gontang et al. [29] used an oligotrophic medium similar to NPS medium, consisting only of seawater and agar, to cultivate Gram-positive bacteria from sediments collected in an intertidal zone in the Republic of Palau, and was able to obtain 70% of the total isolates (199 Actinobacteria and Firmicutes) in this medium.

The selective media SCN and M3 have been also described as efficient media for the isolation of Actinobacteria from marine sources, though in our study SCN only allowed the isolation of a small fraction of Actinobacteria strains, and M3 retrieved no isolates. Maldonado et al. [31] employed, among other culture media, M3 and SCN, for the isolation of Actinobacteria from several marine sediments collected at different locations (Japan Trench, Canary Basin and Norway fjords) and, contrary to our results, they were able to recover a large number of Actinobacteria isolates in these two media (704 isolates). Girão et al. [32] used SCN medium for the isolation of Actinobacteria from *Laminaria ochroleuca* collected in northern Portugal and was able to obtain 54 isolates belonging to *Streptomyces*, *Isoptericola*, *Rhodococcus*, *Nonomuraeae*, *Nocardiopsis*, *Microbispora* and *Microbacterium* genera.

Marine Actinobacteria are a recognized source of bioactive compounds [1,33]. In our study, all Actinobacteria strains isolated were screened for their antimicrobial and cytotoxic activities. Antimicrobial assays showed crude extracts of six Streptomyces isolates active against *C. albicans*. This is not a surprising result as members of this genus are widely recognized for their unique capacity to produce bioactive secondary metabolites with various activities [6,33,34]. The capacity of Streptomyces species isolated from marine sediments to inhibit growth of fungal microorganisms, including *C. albicans*, has been reported before [24,25,26,33]. Examples of some secondary metabolites produced by marine *Streptomyces* with activity against *C. albicans* include isoikarugamycin, a polycyclic tetramic acid macrolactam that is effective at a MIC value of 2–4 μg mL^−1^ [35], daryamides A and B, exhibiting MIC values of 62.5 and 125 μg mL^−1^, respectively [36] and 4-phenyl-1-napthyl-phenyl acetamide, with a strong antifungal activity particularly against *C. albicans* [37]. The comparison of the MIC values of these compounds with those obtained in our study (values in the range of 3.90–125 μg mL^−1^), suggest that the crude extracts of *our Streptomyces* strains exhibit a potent antifungal activity, since the MIC values were obtained for crude extracts and not for pure compounds as those indicated above.

Screening of cytotoxic activity showed that 25 crude extracts of Actinobacteria exhibited significant effects, at the final concentration of 15 µg mL^−1^, on the viability of the tested cell lines (Figure 3). Many of these extracts not only decreased the cellular viability of the cancer cell lines but also had an effect on the nontumor cell line, suggesting general cytotoxicity. However, some crude extracts showed selective anticancer activity in a specific cancer cell line, without affecting the nontumor cell line. The strains that demonstrated cytotoxic activity in our study (*Micromonospora*, *Streptomyces* and *Actinomadura* spp.) are known producers of anticancer compounds. *Micromonospora* species isolated from marine sediments were reported to produce the anticancer compounds diazepinomycin, an alkaloid compound with high antitumor activity, lagumycin B, an angucycline antibiotic with cytotoxicity against diverse cancer and noncancer cell lines, and streptonigrin, a natural compound with potent activity against human neuroblastoma SH-SY5Y cell line [38,39,40]. A vast number of secondary metabolites with anticancer activity has been described for *Streptomyces* strains isolated from marine sediments, such as daryamides, with moderate cytotoxicity against human colon carcinoma cell line, and aureoverticillactam and chromomycin B, exhibiting high cytotoxic activity against several cancer cell lines [36,41,42,43].

*Actinomadura* species retrieved from marine sediments have been also described to produce metabolites with strong anticancer activity, such as ZHD-0501, a staurosporine analogue that was shown to inhibit the proliferation of human lung adenocarcinoma, hepatocarcinoma and leukemia cancer cells, and chandrananimycin A-C, three antibiotics exhibiting activity against several human cancer cell lines [44,45].

Interestingly, strain MS14B, which potentially constitutes a new *Streptomyces* species, showed no noteworthy bioactivities in the tested assays. However, as new phylogenies favor the discovery of novel secondary metabolites, this being especially true for Streptomyces, bioprospection of this strain should be further carried out.

The results obtained in the bioactivity assays indicate the presence of active metabolites in the extracts and, thus, adds a potential for the discovery of new compounds with biotechnological interest. Despite all efforts to discover new NP, there are some issues that lead to considerable time loss such as the recurrent rediscovery of known compounds, the redundant exploration of taxonomically ambiguous microbial strains and the investigation of irrelevant crude extracts in terms of a selected biological application [46]. Consequently, dereplication and molecular networking analyses of Actinobacteria bioactive metabolites were carried out to verify whether the observed activities could result from known compounds. In a first analysis, several compounds from the family of antimycins and nocardamines were found associated with different extracts from *Streptomyces* strains. Antimycin-type compounds are a large and diverse family of natural products produced by Actinobacteria species, including *Streptomyces* strains isolated from marine sediments. This family of compounds has received great interest due to its potent and diverse bioactivities, such as antifungal and anticancer [47]. Nocardamine is part of the super family of siderophore synthetase and is an antibiotic originally produced by a Nocardia strain. Currently, nocardamines have been isolated from other bacterial species, including Actinobacteria from marine sources [48].

In a second analysis, three additional dereplications were performed for eleven clusters. Hits from Actinobacteria or other bacteria were found in seven clusters that correspond to the accurate masses obtained (Figure S1). For the crude extract of *Micromonospora coxensis* MS5B, two different clusters were found, one associated with the ansamycin family (ansatrienin B and benzoxazomycin) and the other with ferrioxamine-based molecules. Ansamycin antibiotics are a group of secondary metabolites produced *by Streptomyces* species, which have a wide range of biological activity [49]. Ferrioxamines, belonging to the group of siderophore compounds, act on the uptake of iron by organisms such as bacteria. These compounds have already been reported as metabolites of *Streptomyces* species and are used in malaria therapy [50]. Three different clusters of the *Micromonospora* sp. MS8B were potentially matched with different compounds produced by the phyla Firmicutes and Proteobacteria. Lichenysin is a lipopeptide biosurfactant with antimicrobial activity, synthesized by *Bacillus* species. Although the production of biosurfactants by microorganisms have been studied in recent years, marine environments are still very scarcely explored in this regard [51]. Ngercheumicins are depsipeptides are produced by marine *Photobacterium* strains [52]. These compounds can interfere with the quorum sensing pathways present in the marine environment and can be supplemented to culture media to promote the growth of slow-growing marine bacteria [53]. The antibiotic Sch 419558 is a lipopeptide isolated from *Pseudomonas fluorescens* and has antibacterial activity against *E.coli* [54]. The aminoglycosides antibiotics, kanamycin and capreomycin were the hits associated with *Streptomyces* sp. MS14B. These compounds were isolated from *Streptomyces kanamyceticus* and *Streptomyces capreolus*, and are used as second-line drugs against multidrug-resistant tuberculosis [55]. The accurate masses of the cluster associated with *Micromonospora aurantiaca* MS55 correspond to possible new versions of niphimycin. This macrolide antibiotic is produced by *Streptomyces* strains and has broad-spectrum antifungal activity [56].

Molecular networking analysis confirmed the presence of four clusters, one from *Micromonospora coxensis* MS52A, two from *Micromonospora* sp. MS8B and one from *Micromonospora* sp. MS56, that likely correspond to new natural products. However, only three of them (M52A, MS8B and MS56), had a relatively high abundance in the crude extract chromatograms, paving the way for future isolation and structure elucidation, which we will pursue. Furthermore, because only clusters associated with a single extract were analyzed in detail, there may be additional chemical novelty in the set of crude extracts that do not show relevant GNPS dereplication.

## 5. Conclusions

This study contributes to increase the scant information available on culturable Actinobacteria associated with marine sediments of the Portuguese coast and on their bioactive potential. Our results suggest that coastal marine sediments can be an important source of Actinobacteria and of novel bioactive natural products and highlight the need of preserving the biodiversity of such environments.

## Figures and Tables

**Table 1:** Actinobacteria isolates obtained in this study with antimicrobial activity. MIC: Minimal inhibitory concentration.

Isolate	Closest Identification	Disk Diffusion Method					
		*E. coli*	*S. typhimurium*	*S. aureus*	*B. subtilis*	*C. albicans*	MIC (µg mL−1)
MS8A1	*Streptomyces* sp.	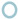	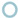	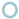	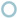	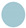	15.62
MS18A	*Streptomyces* sp.	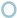	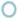	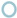	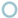	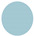	15.62
MS18B	*Streptomyces* sp.	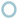	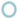	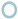	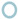	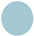	3.90
MS27A	*Streptomyces* sp.	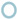	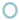	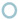	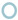	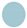	62.5
MS29	*Streptomyces* sp.	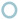	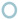	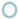	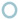	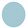	15.62
MS54	*Streptomyces* sp.	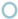	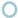	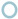	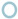	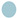	125

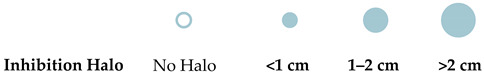

## Supplementary Materials

The following are available online at https://www.mdpi.com/2076-2607/8/11/1691/s1, Table S1: Taxonomic identification of the Actinobacteria strains isolated from the marine sediment collected at Cepães beach in the Parque Natural do Litoral Norte, northern Portugal, and corresponding GenBank accession number. Table S2: GNPS dereplication results for the 26 actinobacterial crude extracts that showed antimicrobial and/or cytotoxic activities. Figure S1: Dereplication of GNPS molecular network data for bioactive actinobacterial extracts.
