# Supplementary material for: Diversity and Bioactive Potential of Actinobacteria Isolated from a Coastal Marine Sediment in Northern Portugal
Source: Microorganisms. 2020 Oct 30;8(11):1691. doi: 10.3390/microorganisms8111691 (PMC7692593; doi:10.3390/microorganisms8111691)
Supplement: Supplementary file 1 [file microorganisms-08-01691-s001.pdf]

Table S1. Taxonomic identification of the actinobacterial strains isolated from the marine sediment collected at Cepães beach in the Parque Natural do Litoral Norte, northern Portugal, and corresponding GenBank accession number.

Strain	Sample treatment	Isolation Medium	Medium used for biomass growth	Closest identification*	Similarity %	Sequence length (bp)	GenBank acession number
**MS3B**	Water bath (60 ºC for 5 min) - Method 2	NPS	Marine Broth	*Micromonospora* sp.	99.78	1361	MN134370
**MS3C**	Antibiotics (20ppm) + Water bath (28ºC for 30 min) - Method 3	NPS	Marine Broth	*Micromonospora* sp.	100	1056	MN134369
**MS3C1**	Water bath (60 ºC for 5 min) - Method 2	NPS	Marine Broth	*Micromonospora* sp.	99.91	1057	MN134368
**MS3D1**	No treatment - Method 1	NPS	Marine Broth	*Streptomyces* sp.	100	1374	MN134350
**MS3E**	Antibiotics (20ppm) + Water bath (28ºC for 30 min) - Method 3	NPS	Marine Broth	*Micromonospora* sp.	99.85	1377	MN134367
**MS5B**	Antibiotics (20ppm) + Water bath (28ºC for 30 min) - Method 3	NPS	Marine Broth	*Micromonospora coxensis*	99.71	1385	MN134380
**MS5C**	Antibiotics (20ppm) + Water bath (28ºC for 30 min) - Method 3	NPS	Marine Broth	*Micromonospora coxensis*	99.71	1388	MN134379
**MS5D**	No treatment - Method 1	NPS	Marine Broth	*Micromonospora coxensis*	99.91	1057	MN134378
**MS5E**	No treatment - Method 1	NPS	Marine Broth	*Micromonospora aurantiaca*	100	1379	MN134392
**MS8A1**	Antibiotics (20ppm) + Water bath (28ºC for 30 min) - Method 3	NPS	ISP2	*Streptomyces* sp.	100	1364	MN134348
**MS8B**	Antibiotics (20ppm) + Water bath (28ºC for 30 min) - Method 3	NPS	Marine Broth	*Micromonospora* sp.	100	1350	MN134362
**MS8C**	Antibiotics (20ppm) + Water bath (28ºC for 30 min) - Method 3	NPS	Marine Broth	*Micromonospora chalcea*	99.85	1377	MN134385
**MS14B**	Antibiotics (20ppm) + Water bath (28ºC for 30 min) - Method 3	NPS	Marine Broth	*Streptomyces* sp.	98.13	1385	MN134356
**MS16B**	Water bath (60 ºC for 5 min) - Method 2	NPS	Marine Broth	*Micromonospora chalcea*	99.85	1376	MN134391
**MS18A**	Water bath (60 ºC for 5 min) - Method 2	NPS	ISP2	*Streptomyces* sp.	99.71	1399	MN134355
**MS18B**	Water bath (60 ºC for 5 min) - Method 2	NPS	Marine Broth	*Streptomyces* sp.	99.85	1376	MN134354

**MS19**	Water bath (60 ºC for 5 min) - Method 2	NPS	Marine Broth	*Micromonospora* sp.	99.57	1386	MN134375
**MS19A**	Water bath (60 ºC for 5 min) - Method 2	NPS	Marine Broth	*Micromonospora chalcea*	100	1354	MN134390
**MS19B1**	Water bath (60 ºC for 5 min) - Method 2	NPS	Marine Broth	*Micromonospora aurantiaca*	100	1357	MN134395
**MS19C**	Water bath (60 ºC for 5 min) - Method 2	NPS	Marine Broth	*Micromonospora* sp.	99.93	1363	MN134374
**MS19D**	Water bath (60 ºC for 5 min) - Method 2	NPS	Marine Broth	*Micromonospora marina*	99.78	1373	MN134377
**MS19E**	No treatment - Method 1	NPS	Marine Broth	*Micromonospora coxensis*	99.85	1377	MN134384
**MS19F**	No treatment - Method 1	NPS	Marine Broth	*Micromonospora chalcea*	99.78	1383	MN134389
**MS23A**	Water bath (60 ºC for 5 min) - Method 2	NPS	Marine Broth	*Micromonospora chalcea*	99.83	1380	MN134388
**MS23B**	Water bath (60 ºC for 5 min) - Method 2	NPS	Marine Broth	*Micromonospora coxensis*	99.78	1381	MN134383
**MS23C**	No treatment - Method 1	NPS	Marine Broth	*Micromonospora* sp.	99.78	1367	MN134373
**MS23D**	No treatment - Method 1	NPS	Marine Broth	*Micromonospora chalcea*	99.85	1377	MN134387
**MS24**	Water bath (60 ºC for 5 min) - Method 2	NPS	Marine Broth	*Micromonospora* sp.	99.71	1379	MN134372
**MS26H**	No treatment - Method 1	NPS	Marine Broth	*Micromonospora* sp.	99.71	1372	MN134361
**MS26I**	No treatment - Method 1	NPS	Marine Broth	*Micromonospora* sp.	99.71	1376	MN134371
**MS27A**	Water bath (60 ºC for 5 min) - Method 2	NPS	ISP2	*Streptomyces* sp.	100	1357	MN134353
**MS29**	Water bath (60 ºC for 5 min) - Method 2	NPS	ISP2	*Streptomyces* sp.	100	1346	MN134352
**MS32**	No treatment - Method 1	NPS	ISP2	*Streptomyces* sp.	99.93	1386	MN134351
**MS35**	No treatment - Method 1	nps	ISP2	*Nocardiopsis* sp.	100	1141	MN134360
**MS38**	Water bath (60 ºC for 5 min) - Method 2	SCN	ISP2	*Herbiconiux sp*	100	1367	MN134396
**MS39B**	Antibiotics (20ppm) + Water bath (28ºC for 30 min) - Method 3	SCN	ISP2	*Arthrobacter pascens*	100	1384	MN134398

**MS39C**	Antibiotics (20ppm) + Water bath (28ºC for 30 min) - Method 3	SCN	ISP2	*Arthrobacter* sp.	100	1059	MN134397
**MS40A**	Antibiotics (20ppm) + Water bath (28ºC for 30 min) - Method 3	SCN	ISP2	*Pseudarthrobacter* sp.	99.25	938	MN134357
**MS40B1**	Antibiotics (20ppm) + Water bath (28ºC for 30 min) - Method 3	scn	ISP2	*Pseudarthrobacter* sp.	99.82	1087	MN134358
**MS42**	No treatment - Method 1	NPS	Marine Broth	*Micromonospora aurantiaca*	100	1382	MN134394
**MS46**	No treatment - Method 1	NPS	Marine Broth	*Micromonospora coxensis*	99.64	1382	MN134382
**MS48**	No treatment - Method 1	NPS	Marine Broth	*Micromonospora* sp.	100	1372	MN134366
**MS49**	No treatment - Method 1	NPS	Marine Broth	*Micromonospora chalcea*	99.63	1358	MN134386
**MS50**	No treatment - Method 1	NPS	Marine Broth	*Micromonospora peucetia*	99.21	1384	MN134376
**MS51**	No treatment - Method 1	NPS	Marine Broth	*Micromonospora* sp.	100	1056	MN134365
**MS52A**	Water bath (60 ºC for 5 min) - Method 2	NPS	Marine Broth	*Micromonospora coxensis*	99.64	1391	MN134381
**MS52B**	Water bath (60 ºC for 5 min) - Method 2	NPS	Marine Broth	*Micromonospora* sp.	100	1360	MN134364
**MS53**	Water bath (60 ºC for 5 min) - Method 2	NPS	Marine Broth	*Polymorphospora rubra*	99.78	1368	MN134359
**MS54**	No treatment - Method 1	NPS	ISP2	*Streptomyces* sp.	99.93	1380	MN134349
**MS55**	Antibiotics (20ppm) + Water bath (28ºC for 30 min) - Method 3	NPS	Marine Broth	*Micromonospora aurantiaca*	100	1376	MN134393
**MS56**	Antibiotics (20ppm) + Water bath (28ºC for 30 min) - Method 3	NPS	Marine Broth	*Micromonospora* sp.	99.85	1377	MN134363
**MS58**	Antibiotics (20ppm) + Water bath (28ºC for 30 min) - Method 3	NPS	Marine Broth	*Actinomadura sputi*	99.39	814	MN134399

*According to Nucleotide collection (nr/nt) database from NCBI BLAST.

Table S2. GNPS dereplication results for the 26 actinobacterial crude extracts that showed antimicrobial and/or cytotoxic activities. The Table shows the compounds recorded in the database for each extract and the correspondent cosine score (indicates the similarity of two MS/MS spectra from 0 to 1, totally dissimilar and completely identical, respectively).

Strain	Closest identification	Compound	Cosine	m/z Error ppm	Lib m/z
**MS3B**	*Micromonospora* sp.	No match	-	-	-
**MS3C**	*Micromonospora* sp.	No match	-	-	-
**MS3C1**	*Micromonospora* sp.	No match	-	-	-
**MS3D1**	*Streptomyces* sp.	Antimycin A2	0.92	11	535.27
		Antimycin A1	0.91	0	557.25
**MS3E**	*Micromonospora* sp.	No match	-	-	-
**MS5B**	*Micromonospora coxensis*	No match	-	-	-
**MS5E**	*Micromonospora aurantiaca*	No match	-	-	-
**MS8A1**	*Streptomyces* sp.	Dehydroxynocardamine	0.97	1	585.36
		Deferrioxamine E	0.95	0	601.36
		Antimycin A1	0.94	0	557.25
		Antimycin A2	0.93	10	535.27
		Antimycin A3	0.92	15	521.26
		Desferrioxamine E	0.86	2	623.34
**MS8B**	*Micromonospora* sp.	No match	-	-	-
**MS8C**	*Micromonospora chalcea*	No match	-	-	-
**MS14B**	*Streptomyces* sp.	No match	-	-	-
**MS16B**	*Micromonospora chalcea*	No match	-	-	-
**MS18A**	*Streptomyces* sp.	Deferrioxamine E	0.96	0	601.36
		Dehydroxynocardamine	0.96	0	585.36
		Antimycin A1	0.94	0	557.25
		Antimycin A2	0.93	10	535.27
		Antimycin A3	0.92	16	521.26
		Desferrioxamine E	0.9	2	623.34
**MS18B**	*Streptomyces* sp.	Dehydroxynocardamine	0.97	0	585.36
		Deferrioxamine E	0.96	0	601.36
		Antimycin A1	0.95	0	557.25
		Antimycin A2	0.93	10	535.27
		Desferrioxamine E	0.91	2	623.34
		Antimycin A3	0.92	16	521.26
**MS19C**	*Micromonospora* sp.	No match	-	-	-
**MS19E**	*Micromonospora coxensis*	No match	-	-	-
**MS27A**	*Streptomyces* sp.	Deferrioxamine E	0.97	0	601.36
		Dehydroxynocardamine	0.95	0	585.36
		Desmethylenylnocardamine	0.95	0	587.34
		Desferrioxamine	0.95	1	561.36
		Antimycin A1	0.94	0	557.25
		Desferrioxamine E	0.93	1	623.34
		Antimycin A2	0.93	9	535.27
		Antimycin A3	0.91	15	521.26
**MS29**	*Streptomyces* sp.	Dehydroxynocardamine	0.97	0	585.36
		Deferrioxamine E	0.96	0	601.36
		Antimycin A1	0.94	0	557.25
		Antimycin A2	0.93	10	535.27
		Antimycin A3	0.92	16	521.26
		Desferrioxamine E	0.92	2	623.34
**MS48**	*Micromonospora* sp.	No match	-	-	-
**MS49**	*Micromonospora chalcea*	No match	-	-	-

**MS50**	*Micromonospora peucetia*	No match	-	-	-
**MS52A**	*Micromonospora coxensis*	No match	-	-	-
**MS54**	*Streptomyces* sp.	Antimycin A2	0.9	10	535.27
		Antimycin A1	0.9	0	557.25
**MS55**	*Micromonospora aurantiaca*	No match	-	-	-
**MS56**	*Micromonospora* sp.	No match	-	-	-
**MS58**	*Actinomadura sputi*	No match	-	-	-

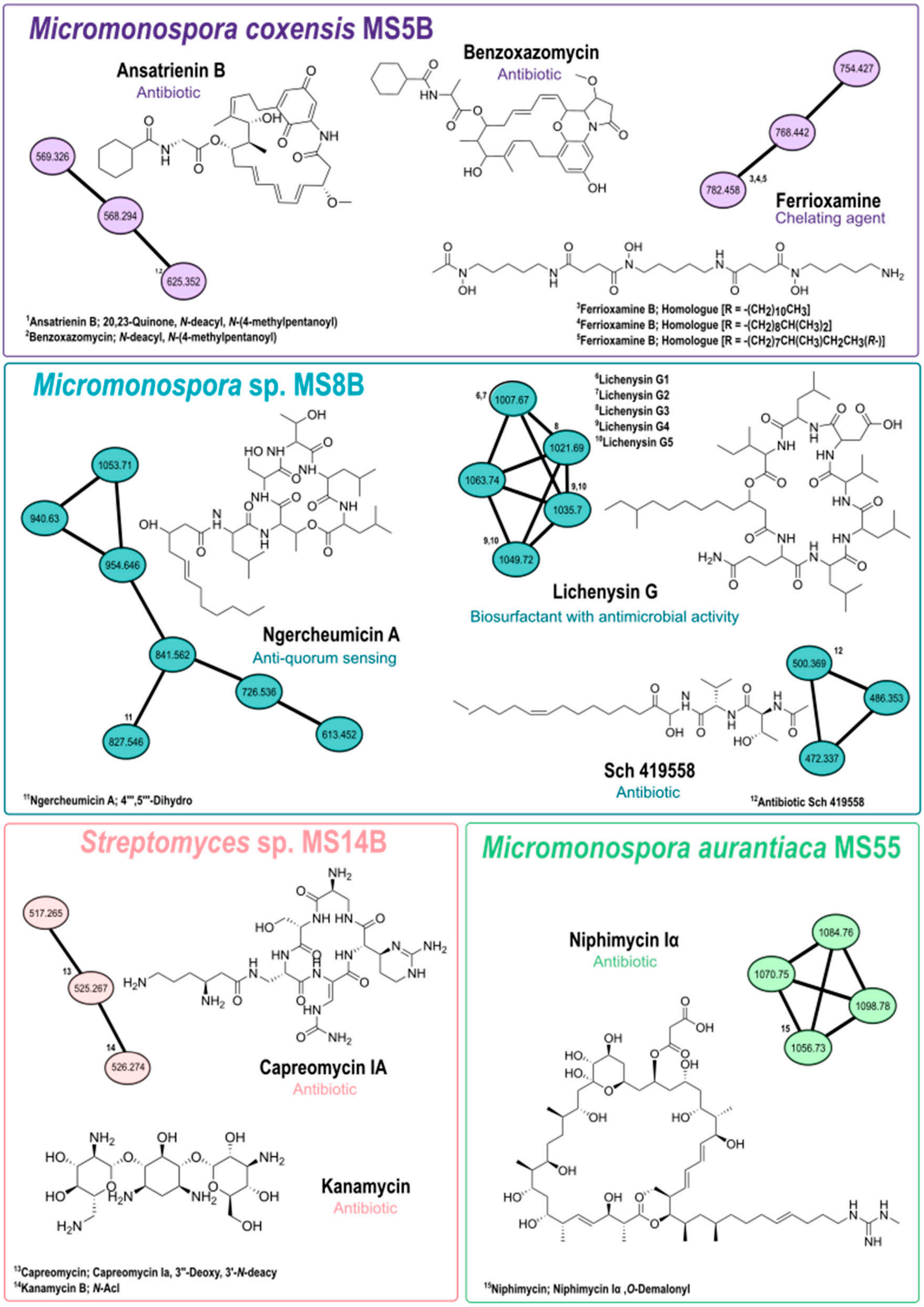

Figure 1. Dereplication of GNPS molecular network data for bioactive actinobacterial extracts. The extracts for the strains MS5B, MS8B, MS14B and MS55 showed clusters with accurate masses matching known natural products, according to the dereplication results from Insilico Peptidic Natural Product Dereplicator, Dictionary of NP and NP atlas database.
